# Retinitis pigmentosa: mutation analysis of *RHO*, *PRPF31*, *RP1*, and *IMPDH1* genes in patients from India

**Published:** 2008-06-14

**Authors:** Mamatha Gandra, Venkataramana Anandula, Vidhya Authiappan, Srilekha Sundaramurthy, Rajiv Raman, Shomi Bhattacharya, Kumaramanickavel Govindasamy

**Affiliations:** 1 Department of Genetics and Molecular Biology, Vision Research Foundation, Sankara Nethralaya Chennai, India; 2 Vitreoretinal Services, Vision Research Foundation, Sankara Nethralaya, Chennai, India; 3 Institute of Ophthalmology, University College London, London, UK

## Abstract

**Purpose:**

To screen for possible disease-causing mutations in rhodopsin (*RHO*), pre-mRNA processing factor 31 (*PRPF31*), retinitis pigmentosa 1 (*RP1*), and inosine monophosphate dehydrogenase 1 (*IMPDH1*) genes in Indian patients with isolated and autosomal dominant forms of retinitis pigmentosa (adRP). Information on such data is not available in India and hence this study was undertaken.

**Methods:**

Blood samples were obtained from 48 isolated and 53 adRP patients, who were recruited for the study. Each patient underwent a detailed clinical examination. Genomic DNA was extracted from the blood samples and screened for mutations in four genes using an ABI3100 Avant genetic analyzer. Reverse transcriptase polymerase chain reaction was performed to amplify the mutated (IVS6+1G/A) mRNA of *PRPF31* in a two-generation adRP family.

**Results:**

Of the 101 probands analyzed, three harbored possible disease-causing mutations. Pathogenic changes were observed in *RHO* and *PRPF31*. A *RHO* mutation, p.Gly106Arg, was found in an isolated RP patient with sectoral RP. Two novel, heterozygous mutations were identified in *PRPF31*: p.Lys120GlufsX122 in an isolated RP patient and a splice site mutation, IVS6+1G/A in an adRP patient. However, no disease-causing changes were observed in *RP1* and *IMPDH1*.

**Conclusions:**

We screened *RHO, PRPF31, RP1*, and *IMPDH1* and identified causative mutations in 4% of isolated and 2% of adRP patients from India. To the best of our knowledge, this is the first report to identify frequencies of mutations in isolated and adRP patients in India.

## Introduction

Retinitis pigmentosa (RP) is a group of inherited retinal degenerative disorders characterized by progressive degeneration of the midperipheral retina, leading to night blindness, visual field constriction, and eventual loss of visual acuity. It is one of the leading causes of blindness in adults with an incidence of around 1 in 3,500 worldwide [1]. Clinical manifestations include pigment deposition in the retina and attenuation of retinal blood vessels followed by atrophy of the retinal pigment epithelium [2]. Electroretinogram (ERG) changes are present with abnormalities of both rod and cone ERGs, but rod ERGs are more affected than cone ERGs [3]. In advanced RP both rod and cone ERG responses are undetectable. RP can be inherited in an autosomal dominant (ad), recessive (ar), or x-linked (xl) manner, as well as in digenic, mitochondrial, or simplex patterns. Most patients with RP are isolated or sporadic with no known affected relatives, although some of these may have inheritances that are autosomal or X-linked recessive or dominant with incomplete penetrance. Despite having similar characteristics, there is a wide spectrum of clinical and genetic heterogeneity between the different modes of inheritance. RP involves nearly 37 genes (Table 1; RetNet). However, this is only a partial representation of the total number of genes since it is believed that more than 60% of RP genes have yet to be identified [4].

**Table 1:** Number of retinitis pigmentosa genes and loci

Mapped and identified genes	*CA4, CRX, FSCN2, GUCA1B, IMPDH1, NR2E3, NRL, PRPF3, PRPF8, PRPF31, PRPH2, RHO, ROM1, RP1, RP9, SEMA4A, TOPORS, ABCA4, CERKL, CNGA1, CNGB1, CRB1, LRAT, MERTK, NR2E3, PDE6A, PDE6B, PRCD, PROM1, RGR, RLBP1, RPE65, SAG, TULP1, USH2A, RP2, RPGR*	37
Mapped loci (not identified)	RP33, RP22, RP25, RP28, RP29, RP32, RP6, RP23, RP24, RP34	10
Total		47

Tremendous research in the field of RP allowed the identification of nearly 47 genes. However, mutations in 37 genes are known to cause adRP and 10 adRP genes have been mapped but not identified yet (RetNet).

According to various reports, adRP represents between 15% and 35% of all RP cases. These values were derived from different studies, with the highest value being found in the United States [5] and the lowest in southern Europe [6]. However, accurate data on the frequency of RP in India is not available. To date, about 17 genes have been identified as causative of adRP. Rhodopsin (*RHO)* is the most frequently reported adRP gene, contributing to 20%–25% of cases [7], followed by pre-mRNA processing factor 31 (*PRPF31*) (2%–8% of adRP cases) [8,9], retinitis pigmentosa 1 (*RP1*) (5%–10%) [10], and inosine monophosphate dehydrogenase 1 (*IMPDH1*) (5%–10%) [11]. The frequency of mutations in these genes has been documented for many populations around the world, but not for patients from India.

All of the aforenamed genes, excluding *PRPF31*, have been reported as disease-causing for arRP. *IMPDH1* not only causes adRP but also Leber congenital amaurosis (LCA) [12]. Currently, treatment for RP is unavailable; however, animal models for a variety of retinal degenerations have been successfully rescued, using gene therapy approaches. Based on this success, it is anticipated that such treatments may become available for patients. Therefore, we undertook the first mutational analysis of four most frequently reported adRP genes in our patients.

## Methods

Study participants were selected after clinical examination (retina clinic) and pedigree analysis (genetic clinic) at Sankara Nethralaya. Clinical tests included Humphrey perimetry, fundus photography, and electroretinogram (ERG). A detailed pedigree was taken from the probands. Criteria for selecting adRP families were based on the occurrence of at least two affected generations with both sexes along with evidence of male to male transmission. The details of the patients and controls participated in the current study are given in Table 2. Genomic DNA was extracted from each 3 ml blood sample collected from 48 isolated and 53 adRP patients and 75 unrelated controls using standard phenol-chloroform methodology. Informed consent was obtained from all participants, and the research adhered to the tenets of Declaration of Helsinki and was approved by the hospital Internal Review Board. All the coding regions and the adjacent flanking intronic sequences of *RHO, PRPF31,* and *IMPDH1* and the exons harboring previously reported mutations of the *RP1* gene, namely exons 4F, 4G, and 4H of *RP1* were amplified by polymerase chain reaction (PCR). The primers and the amplification conditions used were as reported previously [11,13-15]. PCR amplified products were electrophoresed at 100 v for 30 min on 2% ethidium bromide-agarose gels. The ethidium bromide-stained gels were captured by the gel documentation system ImageMasterR VDS (Amersham Pharmacia Biotech, Piscataway, NJ) using the Liscap software and analyzed using the Imagemaster Totallab gel documentation system. The PCR products were directly sequenced with ABI 3100 Avant Genetic Analyzer (Applied Biosystems, Foster City, CA) using the same oligomers employed in the PCR reactions. The patients' sequences were compared to known *RHO* (GenBank NM_000539), *PRPF31* (GenBank NM_015629), *RP1* (GenBank NM_006269), and *IMPDH1* (GenBank NM_000883) sequences.

**Table 2:** Details of the participants in the study.

**Participants**	**Gender**		**Average age (years)**	**Diagnosis**
	**Male**	**Female**		
Isolated	32	16	35 (range 12-60 years).	RP
adRP	28	25	30.5 (range 3-61years)	RP
Controls	50	25	61.5 (range 42-70 years)	normal

Of 151 individuals recruited for the study, 101 were RP patients (48 isolated and 53 adRP) and 50 were unrelated healthy individuals. All the patients demonstrated typical features of RP. The average age has been calculated for all three groups of participants and is as tabulated above. However to overcome the problem of late onset of the disease in control group and to ensure the presence of the normal individuals in the same, we recruited individuals of age group between 42-70 years.

### Reverse transcriptase-polymerase chain reaction reactions

RNA was isolated from 10 ml of heparinized blood samples, for each of the five members of an adRP family (family A) having the *PRPF31* (IVS6+1G/A) mutation. RNA isolation was done using Trizol reagent (Sigma-Aldrich, St. Louis, MO), according to the manufacturer’s instructions. Blood (10 ml) was collected from each individual. DNase-treated RNA, cloned MMLV reverse transcriptase (USB, Cleveland, OH) and random hexamers (Amersham Biosciences, Piscataway, NJ) were used to generate cDNA by reverse transcription-polymerase chain reaction (RT–PCR). The transcribed *PRPF31* fragment from exon 3 to exon 8 was amplified using the following paired primers (forward, 5'-AAG TGA TGG GAC CAG TGG AG-3'; reverse, 5'-GTA GAC GAG AAG CCC GAC AG-3') and the conditions previously reported [16].The thermocycling profile was as follows: RT reaction (50 °C for 60 min, 80 °C for 2 min), followed by a three-step touchdown PCR (94 °C for 50 s; 65 °C for 1 min, drop 1 °C per cycle; 72 °C for 1 min) for 10 cycles, followed by a three-step PCR (94 °C for 50 s, 58 °C for 1 min, 72 °C for 1 min) for 24 cycles.

## Results

Disease-causing mutations were identified in three out of 101 unrelated RP patients screened. We observed a heterozygous p.Gly106Arg mutation in *RHO* as well as two heterozygous mutations in *PRPF31*: p.Lys120GlufsX122 and c.527+1G/A. None of these changes were found in 75 unrelated healthy controls.

### *RHO* screening

#### p.Gly106Arg (c.316G/A)

**Figure 1:**
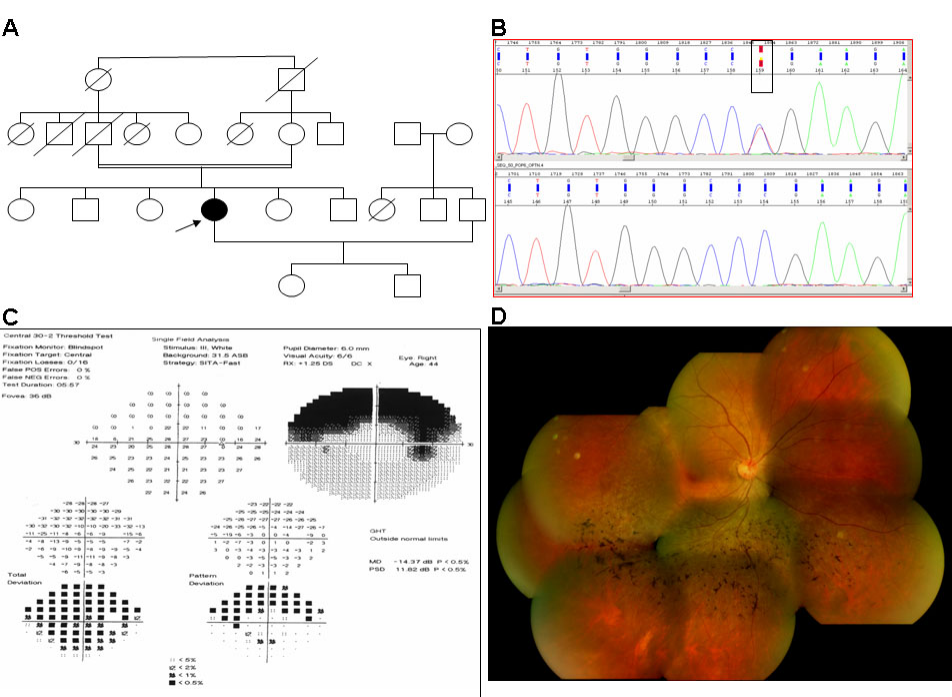
Clinical and molecular features of the proband (L2:50) with the p.Gly106Arg mutation. **A:** Pedigree showing the isolated form of the disease. **B:** Genomic DNA sequences (reverse) of a part of the *RHO* gene of L2:50 displaying the p.Gly106Arg mutation (top) and of a normal subject (bottom). The rectangular box shows the position of a heterozygous change at nucleotide 316 (c.316G/A, but the sequence shows the reverse sequence boxed as Y). **C:** Visual field test reveals a sectoral form of RP. **D:** Fundus (right eye) photograph showing mild retinitis pigmentosa changes.

A missense change, p.Gly106Arg was identified (Figure 1) in an isolated RP patient (L2:50). However this change was not seen in any of the other family members or in 75 unrelated controls. Two intronic changes, c.232+4C/T and –26 A/G, were also detected in the study group.

L2:50, a 44-year-old woman from southern India, had a two-year history of visual disability. She also had a history of night blindness for 1^1^/_2_ years but had no history of night blindness in her family. Upon examination, her vision was 6/9; N6 in the right eye and 6/12^+2^; N6 in the left eye. Vision improved with – 0.50 DC X 90° to 6/6^−1^ and in the left eye –0.50 DS / – 0.50 DC X 120° to 6/6. Near vision was N6 with +1.50 DS. She was orthophoric; ocular movements were full, free, and painless. Slit-lamp examination revealed no abnormality except early posterior subcapsular cataract in both eyes. Intraocular pressure with applanation tonometry was 18 mmHg in the right eye and 17 mmHg in the left eye. Fundus examination with indirect ophthalmoscopy after full dilatation revealed a normal disc. There were sectoral lesions of bony corpuscles seen in the inferotemporal quadrant of both eyes .The patient was given the clinical diagnosis of bilateral sectoral RP. Humphrey visual field examination was done in both eyes. It showed superior constriction of the fields corresponding to inferior sectoral RP. ERG showed reduced rod and cone response in both eyes consistent with sectoral RP. None of the other family members were affected.

### *PRPF31* screening

We identified two novel pathogenic changes: 1) p.Lys120GlufsX122 (GenBank DQ383415) in an isolated RP case (N1:51); and 2) IVS6+1G/A (GenBank DQ374434) in an adRP family (family A). Five nonpathogenic intronic variations (Table 3) were also identified in the study group.

**Table 3:** Isocoding changes and polymorphisms identified.

**Gene**	**Variations**	**Location**	**Frequency**
*RHO*	c.232+4C/T	Intronic	3^ 1* 2$
	–26 A/G	Intronic	1^ 1* 2$
*PRPF31*	c.238+93T/C	Intronic	4^ 2* 3$
	c.322+21G/A	Intronic	3^ -* 1$
	c.420+81T/C	Intronic	12^ 8* 6$
	c.420+82C/G	Intronic	9^ 14* 4$
	c.499+55G/A	Intronic	1^ -* 2$
*RP1*	p. Arg 872 His	Intronic	27^ 20* 14$
*IMPDH1*	c.383+33C/T	Intronic	1^ 1* 1$
	c.383+54delG	Intronic	3^ 1* 2$
	p.Leu244Leu	Coding	13^ 7* 5$
	p.Gln427Gln	Coding	5^ 1* 2$
	p.Ala440Ala	Coding	1^ -* 1$

The polymorphisms identified in *RHO, PRPF31*, and *RP1* were found in the noncoding or intronic regions. However, in *IMPDH1*, out of 5 polymorphisms, two were in the intronic region and the remaining three were in the exonic region (isocoding changes). Abbreviations: The "^" symbol denotes isolated, the "*" denotes adRP and the "$" symbol denotes controls.

#### p.Lys120GlufsX122 (c.358_359 del AA)

**Figure 2:**
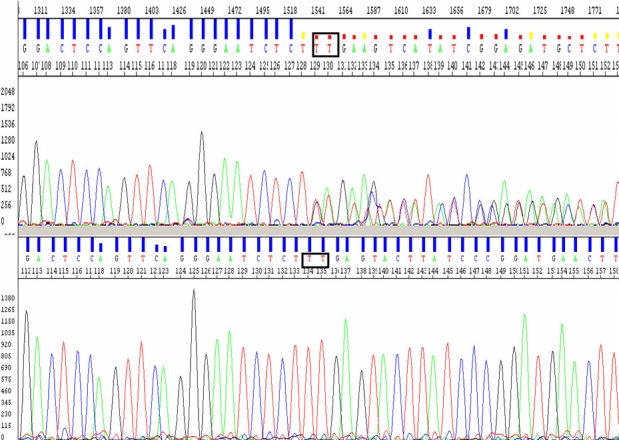
Electropherograms displaying a novel *PRPF31* mutation, p.Lys120GlufsX122. Genomic DNA sequences (reverse primer) of a part of *PRPF31* from a patient with the p.Lys120GlufsX122 mutation (top) and from a normal subject (bottom). The rectangular box shows the position of a heterozygous deletion of two nucleotides at codon 120 (c. c.358_359 del AA, but the sequence shows the reverse sequence boxed as TT).

The deletion of two nucleotides AA at codon 120 in exon 5 (Figure 2) of *PRPF31* leading to a frameshift was observed in patient N1:51. The truncated protein consisted of 122 amino acids with three novel amino acids before the stop codon. The patient, N1:51, could not be contacted, therefore detailed clinical examination of the proband and genetic analysis for the N1:51 family could not be performed.

#### IVS6+1G/A (c.358_359del AA)

**Figure 3:**
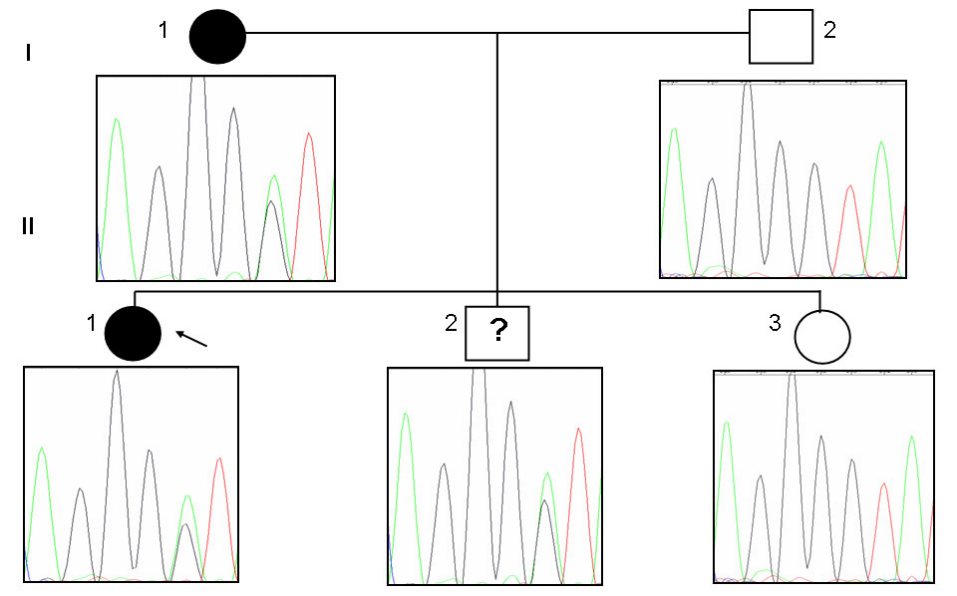
Pedigree of family A harboring a IVS6+1G/A mutation in *PRPF31.* The change in the genomic DNA sequence was observed in two affected patients and an asymptomatic individual. Normal individuals are shown as clear circles (females) or squares (males), and affected individuals are shown as solid symbols. The clear square with the symbol “?” indicates an asymptomatic carrier.

**Figure 4:**
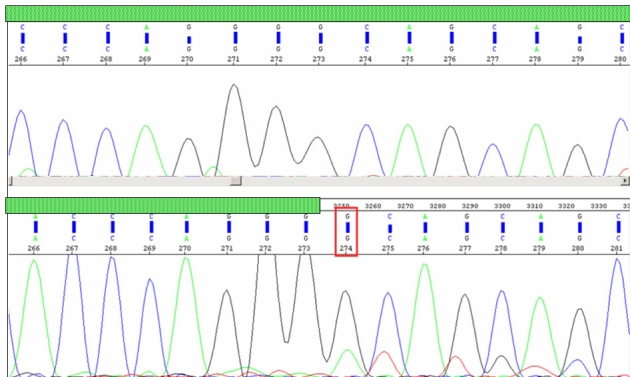
Electropherograms displaying intron 6 retention. A comparison of a portion of *PRPF31* cDNA sequences between the unaffected (top: I-2) and the affected (bottom: II- 1) revealed intron 6 retention in all affected and asymptomatic individuals of family A. The green hatched box represents exonic region, and the red rectangular box indicates the start position of intron 6 retention (non-hatched) in the proband.

**Figure 5:**
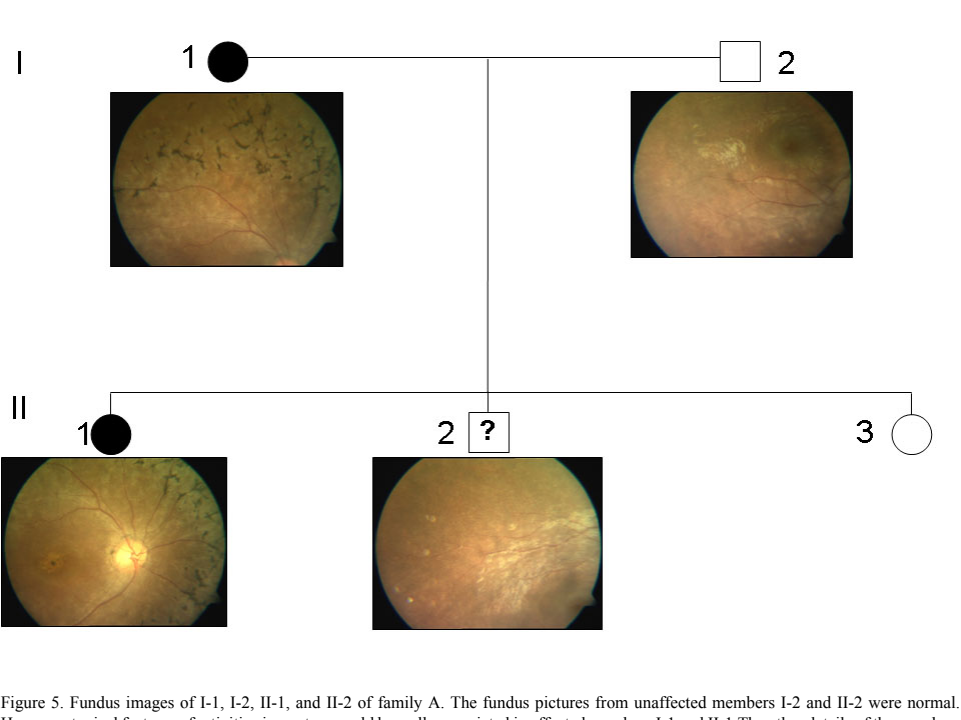
Fundus images of I-1, I-2, II-1, and II-2 of family A. The fundus pictures from unaffected members I-2 and II-2 were normal. However, typical features of retinitis pigmentosa could be well appreciated in affected members I-1 and II-1.The other details of the members of family A are as follows; Individual I-1 was a 24-year-old female with a history of night blindness since 8 years. Her visual acuity was counting fingers at 4 meters, and was not improving with glasses (NIG). Fundus examination revealed arteriolar attenuation, bony spicules with degenerative macular changes, and disc pallor. The electroretinogram (ERG) was nonrecordable, fields were grossly defective. Individual, I-2 was a 32-year-old male with a vision of 6/6 and normal fundus. Individual II-1 (proband) was a 5-year-old female with a complaint of night blindness since 6 months. Fundus revealed attenuated vessels, normal disc, dull foveal reflex, and altered retinal sheen. The ERG was nonrecordable in both the eyes. Individuals II-2 was a 3.5-year-old male with normal vision and fundus. Individual II-3 is a 2-year-old female with normal fundus. The ERG and visual field test could not be done due to pediatric age.

The proband with this splice site mutation was the child of an affected mother and unaffected father. Upon further analysis of genomic DNA of other members of family A (Figure 3), we found the mutation in affected members, I-1 and II-1, and a clinically normal individual, II- 2, but not in other two clinically normal individuals, I-2 and II-3. Individual II-2 may not have manifestation for two reasons: either the child was too young (3.5 years) to manifest the disease, or there was incomplete penetrance. To test whether the splice-site mutation leads to a defective mRNA, we performed RT–PCR to amplify *PRPF31* cDNA from total RNA isolated from peripheral blood obtained from all members of family A. A fragment of about 559 bp was obtained, and, as shown in Figure 4, the cDNA sequencing revealed intron 6 retention in two affected members (I-1, II-1) and an asymptomatic individual (II-2). The transversion G/A occurred in the first nucleotide of intron 6, interrupting its splicing and leading to a frameshift and truncated protein of 186 amino acids with 11 novel amino acids before a premature stop. Figure 5 shows fundus photographs documenting four members of family A. The youngest sibling (II-3) underwent an indirect ophthalmoscopic examination and was found to have a normal fundus. However other tests; field of vision, fundus fluorescence angiography and ERG could not be performed in individual II-3 due to very young age.

### *RP1* and *IMPDH1* screening

Except for a few nonpathogenic variations (Table 3), no other variations were observed in *RP1* and *IMPDH1*. All intronic polymorphisms encountered in this study were analyzed using splice site prediction software. It was found that none of these changes were likely to alter RNA splicing.

## Discussion

In our screening of *RHO, PRPF31, RP1,* and *IMPDH1* genes in 101 index patients, we identified three pathogenic changes: one in *RHO* and two in *PRPF31*. In the United States, the United Kingdom, and Europe, *RHO* mutations account for 20%–25% of all adRP cases [7]. So far, only limited research has been performed in India on the genetics of RP hence such data was not available.

Prior to this study, only two RP-related studies have been reported using families from India [17,18]. However these studies did not report the frequency rate of *RHO* mutations in isolated/adRP patients. Kumaramanickavel et al. [17] identified an *RHO* mutation in a family with arRP, whereas Dikshit et al. [18] mainly focused on the mutations reported in codons 345 and 347. In the latter study, the whole gene was not screened and 100 Indian RP patients were recruited from 76 families, irrespective of the inheritance of RP [18]. However, in the present study, a p.Gly106Arg mutation was detected in approximately 2% of isolated RP cases thus suggesting low frequency of *RHO* mutations in the Indian population. The observed low frequency of *RHO* mutations in the current study could be due to ethnic variation or small sample size. However since single gross deletions have also been encountered at a frequency of 0.8% in *RHO*, the direct sequencing based method in the current study would have failed to detect this type of mutation. Another finding is that, similar to previous reports, the same missense mutation was identified in an isolated RP patient, who clinically displayed the sectoral form of RP [19,20].

**Figure 6:**
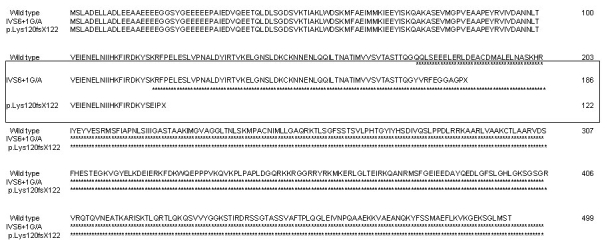
Alignment of protein sequences of the PRPF31 protein The wild-type sequence and two identified novel mutants, IVS6+1G/A and p.Lys120GlufsX122, are shown. Both the mutations resulted in a premature truncation of the protein.

Two novel disease-causing changes were identified in *PRPF31*. Although our sample included patients from all over India, *PRPF31* mutations were identified only in the patients from the northern region of the country. The frameshift mutation, p.Lys120GlufsX122, was identified in an isolated RP patient. However, as the proband was not available for further analysis, we could not assess the penetrance status of the disease in the family. The second novel mutation identified was a splice site mutation, IVS6+1G/A. Upon further genetic analysis of other family members of the proband with IVS6+1G/A, we found the mutation in clinically affected as well as in a clinically normal individual, suggesting incomplete penetrance of the disease in this pedigree. Normally the process of splicing occurs at the first nucleotide of an intron that removes the noncoding region. However, in this case splicing at the first nucleotide of intron 6 was skipped due to the transversion of G/A, thus resulting in the retention of intron 6. Intron retention was confirmed by sequencing cDNA derived from lymphocyte RNA. The introduction of novel nucleotides in the coding region altered the open reading frame, resulting in the premature termination of the protein with a total of 186 amino acids instead of in the wild-type of 499 amino acids (Figure 6); this change was a novel finding.

The contribution of the *PRPF31* mutations in our population was determined to be 2% in isolated cases and 2% in adRP cases. The percentage would increase to 4% in adRP, considering that the isolated RP patient with p.Lys120GlufsX122 could be an adRP case showing incomplete penetrance. In such a case, the frequency of *PRPF31* mutations in adRP was found to be approximately equal to that reported in the United Kingdom (5%) [21], but it is more than what has been reported in Spain (2%) [22], and Japan (2%) [23]. However, the frequency of these mutations are relatively low when compared to reports from the United States (8%) [9]. This could be due to the presence of large deletions, insertions, or genomic rearrangements, which could not be detected by the current techniques employed in this study.

**Figure 7:**
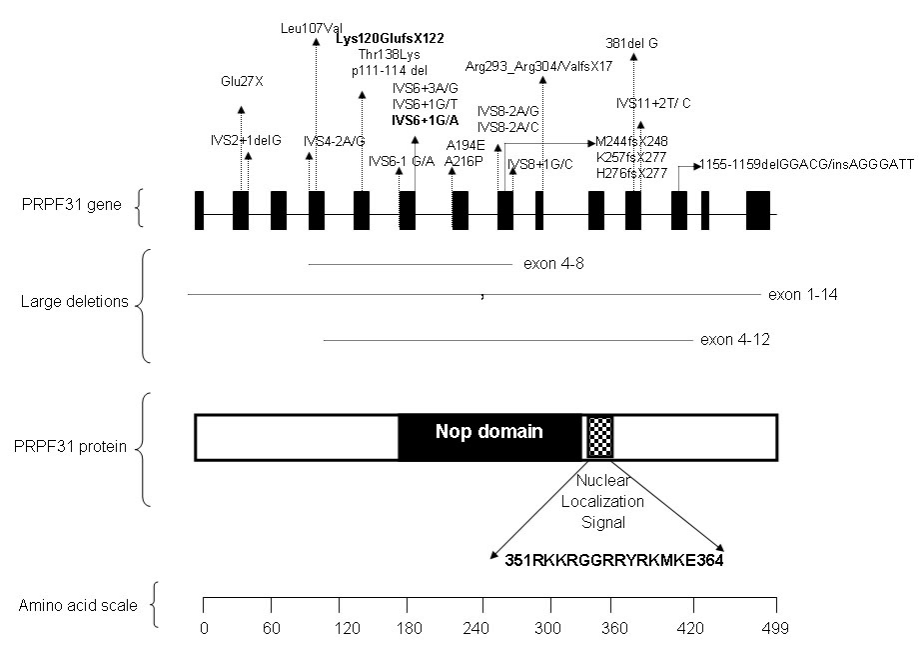
Schematic representation of *PRPF31* and its protein structure. Illustrated are positions of reported mutations (including large deletions) in human *PRPF31* and domain features of predicted PRPF31 protein. Two novel *PRPF31* mutations reported in this study are depicted in bold. Distance between exons and domains are not drawn to scale.

The two novel mutations identified in this study result in the production of protein devoid of nuclear localization signal (NLS; Figure 7). NLS is located between residue 351–364, and it is critical for the export of PRPF31 protein from the cytoplasm to nucleus. Since NLS is absent in both the mutant proteins, the translocation of the protein from the cytoplasm into the nucleus is hindered, affecting its spicing function [24]. Thus, these mutations seem to induce a pathogenic mechanism by haploinsufficiency rather than by a dominant negative effect.

*RP1* screening revealed a previously reported polymorphism, p.Arg 872 His. Pathogenic changes were not identified, due to partial screening of the gene. In this study, we screened only a segment of *RP1* comprising of exons 4F, 4G, and 4H because of the occurrence of frequently reported mutations, p.Arg677X, p.Gln679X, and two other deletions at codon 765 and 763 in this region. Disease-causing changes in *IMPDH1* were not identified, which implies that *IMPDH1* mutations may not be a causative of adRP in our population.

In summary, we identified three pathogenic changes: p.Gly106Arg in *RHO*, and two novel mutations, p.Lys120GlufsX122 and IVS6+1G/A in *PRPF31*. Causative mutations were identified in approximately 3% of our study group. The relative contribution of each gene to the total number of mutations was estimated as 1% *RHO* and 4% *PRPF31*. However, we did not identify causative mutations in *RP1* and *IMPDH1* .To the best of our knowledge, this is the first study undertaken to determine the contribution of commonly reported adRP genes, *RHO*, *PRPF31*, *RP1,* and *IMPDH1* in RP patients from India.
